# Investigation on Microsheet Metal Deformation Behaviors in Ultrasonic-Vibration-Assisted Uniaxial Tension with Aluminum Alloy 5052

**DOI:** 10.3390/ma13030637

**Published:** 2020-01-31

**Authors:** Chunju Wang, Weiwei Zhang, Lidong Cheng, Changqiong Zhu, Xinwei Wang, Haibo Han, Haidong He, Risheng Hua

**Affiliations:** 1 School of Mechanical and Electrical Engineering, Robotics and Microsystems Center, Soochow University, Suzhou 215131, China; ldcheng@suda.edu.cn (L.C.); hdhe@suda.edu.cn (H.H.); 20195229040@stu.suda.edu.cn (R.H.); 2 School of Materials Science and Engineering, Harbin Institute of Technology, Harbin 150001, China; 3 Institute of Electronic Engineering, China Academy of Engineering Physics, Mianyang 621999, China; zhangweiwei0509103@163.com; 4 Laboratory for Space Environment and Physical Sciences, Harbin Institute of Technology, Harbin 150080, China; 5 Shanghai Institute of Spacecraft Equipment, Shanghai 200240, China; haibosmile@163.com

**Keywords:** ultrasonic vibration, acoustic softening, residual effect, microthin sheet, forming limit

## Abstract

Ultrasonic vibration (UV) is widely used in the forming, joining, machining process, etc. for the acoustic softening effect. For parts with small dimensions, UV with limited output energy is very suitable for the microforming process and has been gaininf more and more attention. In this investigation, UV-assisted uniaxial tensile experiments were carried out utilizing GB 5052 thin sheets of different thicknesses and grain sizes, respectively. The coupling effects of UV and the specimen dimension on the properties of the material were analyzed from the viewpoint of acoustic energy in activating dislocations. A reduction of flow stress was found for the existing acoustic softening effects of UV. Additionally, the residual effects of UV were demonstrated when UV was turned off. The uniform deformation ability of thin sheet could be improved by increasing the hardening exponent with UV. The experimental results indicate that UV is very helpful in improving the forming limit in microsheet forming, e.g., microbulging and deep drawing processes.

## 1. Introduction

As demands on miniature/micrometal products increase significantly, for example, metallic parts with microchannels fabricated through ultra-thin sheets are widely used in mass and heat transfer areas, e.g., heat exchangers and proton exchange membrane fuel cells (PEMFCs). Due to its advantageous characteristics for mass production, microforming has become an attractive option in the manufacture of these products [1]. However, the geometrical size of the workpiece, the microstructural length scale of deforming materials, and their interaction significantly affect the deformation response of microscale objects, e.g., flow stress, springback, forming limit. [2,3,4]. Several kinds of microsheet forming processes, e.g., rigid stamping, hydroforming, and rubber pad forming, have been carried out to manufacture microchannels in recent years [5,6]. Microrigid stamping was a commonly used technology for microchannels due to its advantages of low cost and high productivity. A significant thinning easily occurred at the corner regions of the formed microchannel in rigid stamping, which was associated with the tensile force applied at the corner and the friction, resulting in the occurrence of localized strain [7,8]. Then, forming limit diagrams were used to determine the safe limit of the metallic bipolar plates [9]. Since hydroforming has obvious merits, e.g., flexibility, higher drawing ratio, good surface quality, less springback, and the low cost of mold, it has attracted more attention [10,11]. However, the channel height of a hydroformed workpiece was a function of the pressure, and high pressure and the seal became a big problem [5,12]. For rubber pad forming, only half of a single rigid tool is needed, and the punch-cavity misalignment problem can be avoided. A metallic bipolar plate was successfully fabricated using stainless steel SS304 [13] and titanium with a TiN layer [14]. To improve the filling percentage, thinning percentage, and dimensional accuracy, semi-stamp rubber forming was carried out instead of convectional rubber forming [15]. However, the main drawback of the rubber pad forming process is that the life of the rubber pad is not so long and should be replaced after the production of only about 100 plates. Previous investigations have indicated that the existing process cannot be used for the manufacture of microchannels due to the obvious drawbacks mentioned above.

To improve the formability of thin sheet, industrial applications of dynamic load/ultrasonic vibration (UV) were utilized in many metallic sheet forming processes. The application of 20 and 28 kHz oscillation increased the limit drawing ratio (LDR) from 2.68 to 3.01 for cold rolled steel for deep drawing. Greater accuracy and deeper cups can be formed by stopping the oscillation after the maximum punch load rather than applying the oscillation throughout the deep drawing process [16]. In the rigid bulging of microchannels, a kind of dynamic load with a frequency of 0.5Hz could improve the forming depth of 7% and decrease material thinning from 10.7% of static loading to 7.8% [17,18]. In UV-assisted microdeep drawing, the punch force decreased as the oscillation amplitude increased, and LDR increased from 1.67 to 1.83, from 1.75 to 1.92, and from 1.83 to 2 for a thickness of 50, 75, and 100 µm, respectively [19]. Further, wrinkling and cracking could be avoided through ultrasonic vibration to decrease the coefficient of friction between the sheet material and the die [20]. Using UV to form molten plastic as a flexible punch, a trapezoidal cross-section microchannel of 697.2 µm width and 248.4 µm depth was successfully replicated up to 98% on a thin T2 copper sheet of an initial thickness of 50 µm [21,22]. The UV-assisted microforming processes have shown that it was helpful in improving the quality of microparts. However, the mechanism of deformation behavior should be investigated in detail, especially for the thin sheet when UV is applied.

One of reasons dynamic load/ultrasonic can improve formability is the acoustic softening effect, as shown in many research studies. High frequency vibration was found to significantly reduce the apparent static shear stress necessary for the plastic deformation of metals [23]. Further, the amount of reduction was directly proportional to the acoustic energy input to the specimen; this yielding of metals due to ultrasonic irradiation has the possibility to be more efficient than other methods, e.g., heating [24]. To realize the mechanism of the softening effect of ultrasonic vibration, a significant reduction in subgrain formation was found and analyzed from the viewpoint of acoustic energy, which can be attributed to its ability to enhance dislocation dipole annihilation to cause dislocations to travel longer distances [25]. A modeling framework for acoustic plasticity was proposed to model the acoustic softening considering the acoustic energy intensity based on the crystal plasticity theory [26]. An opposite result by a dislocation dynamics simulation indicated that the acoustic effect was associated with extensive enhancement of subgrain formation [27]. However, the effects of UV on the properties of materials have not been investigated considering dimensions of microparts in UV-assisted microforming processes. The mechanism of stress reduction was investigated, and the evolution of the microstructure was observed through experiments and simulation. However, research was seldom carried out on the small specimens. From the viewpoint of acoustic energy, the density is changed during the miniaturization of specimens. Additionally, the density of microdefects in metallic specimens, e.g., dislocations and grain boundaries, becomes smaller in small specimens, which is considered to absorb the acoustic energy. Investigations should be done to analyze the effect of grain size and specimen size on deformation behavior in UV-assisted deformation.

In the investigation, UV-assisted uniaxial tensile tests were carried out utilizing GB 5052 thin sheets with different thicknesses from 50 to 100 μm. Further, the effects of UV parameters, e.g., vibration amplitude and duration, were also studied. Properties of materials, e.g., yield stress, tensile strength, elongation, and hardening exponent, were obtained from the UV-assisted experiments. The coupling effects of UV and specimen dimension were analyzed from the viewpoint of acoustic energy in activating dislocations.

## 2. Materials and Methods

### 2.1. Experimental Set-Up

**Figure 1:**
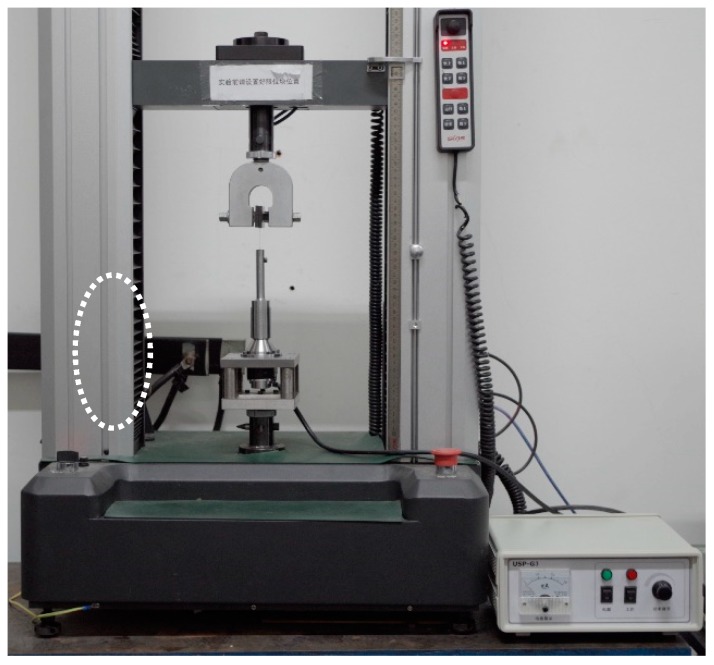
Ultrasonic-vibration-assisted uniaxial tension device.

In this investigation, a kind of ultrasonic-vibration-assisted uniaxial tension device was utilized as shown in Figure 1 [28], which was developed by our group based on a testing machine (UTM6104, Shenzhen Suns Technology Stock Co., Ltd., Shenzhen, China) with a 10 kN load cell. A vibrator was designed, including an ultrasonic transducer and an ultrasonic horn. A piezoelectric ceramic transducer (PZT-8) with a sandwich structure was selected as the ultrasonic transducer, and it could effectively transform the electric energy to mechanical longitudinal vibration. To clamp the thin sheet specimen, a groove structure was designed at the top end of the ultrasonic horn. The width and depth of the groove were selected as 0.2 and 10 mm, respectively, based on finite element analysis using the ABAQUS commercial software (v6.12, Dassault Systems SIMULIA, France). One side of the thin sheet specimen is clamped by the vibrator using the groove structure at the top end of the ultrasonic horn, which is fixed at the bottom parts of the testing machine. Another side of the specimen is clamped by a tool installed on a moving beam of the testing machine. In the uniaxial tension device, the moving beam can move upward, then a force is applied on the specimen to fix the bottom part of the specimen. During the tensile test, the vibrator can be turned on, and UV is applied on the specimen by the ultrasonic horn.

Measured by the laser vibrometer Polytec OFV-5000, the longitudinal vibration frequency of the manufactured vibrator was 21.1 kHz, and its vibration amplitude could be changed from 0.48 to 15 μm depending on the input current, which could meet the requirement of uniaxial tension in these experiments. 

### 2.2. Experimental Material

**Figure 2:**
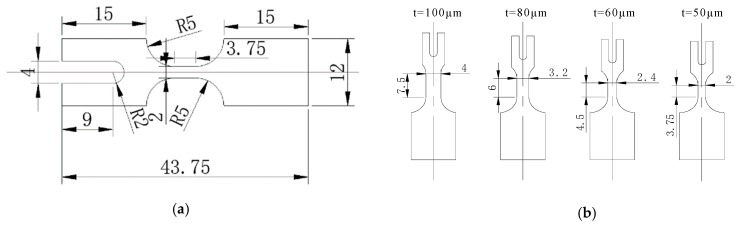
GB 5052 thin sheet specimens and their dimensions. (**a**) Dimensions of specimen (50 μm in thickness); (**b**) Dimensions of specimens with different thicknesses.

**Figure 3:**
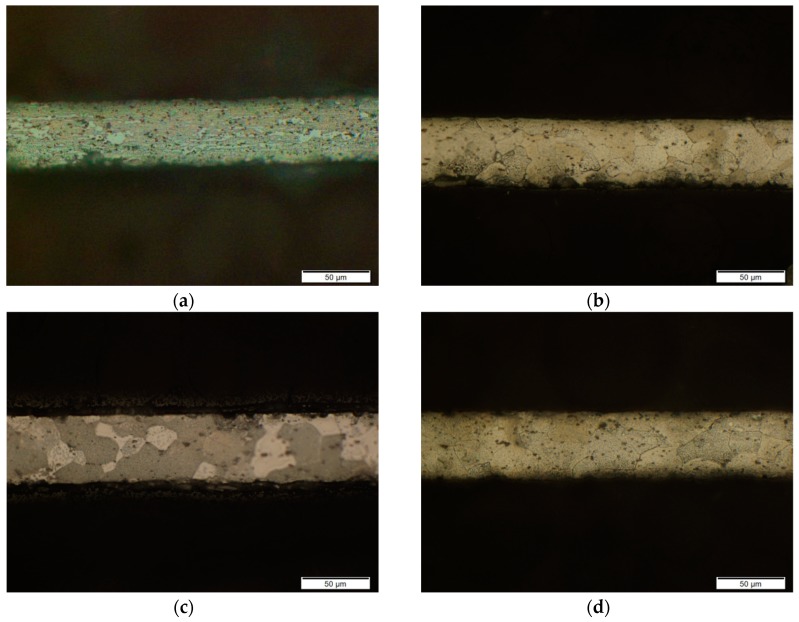

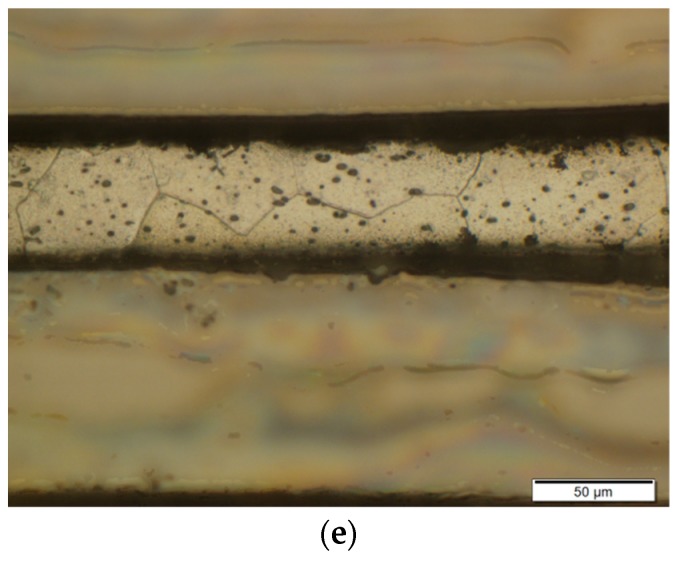
Microstructure of a GB 5052 thin sheet after heat treatment at different temperatures (50 μm in thickness). (**a**) 200 °C; (**b**) 300 °C; (**c**) 400 °C; (**d**) 500 °C; (**e**) 600 °C.

Commercial rolled aluminum alloy GB 5052 sheet was selected in this investigation for its excellent properties, e.g., higher strength alloys, excellent corrosion resistance, and high fatigue strength. The thicknesses *t* of selected rolled aluminum alloy sheets are 50, 60, 80, and 100 μm, and the dimensions of their specimens were designed according to the similar theory as shown in Figure 2. To study the effect of the grain size and reduce the effect of strain hardening induced by the rolling process, heat treatment experiments for a thickness of 50 μm were carried out using a tubular vacuum heat treatment furnace (T1200) at temperatures of 200, 300, 400, 500, and 600 °C for 1 h of the holding time with 10 °C/min of the heat rate, respectively. After heat treatment, specimens were electrochemically polished under a voltage of 5 V and current of 0.5 A for 8 s using a solution of 10 mL of HClO_4_ and 60 mL of C_2_H_5_OH. The obtained microstructures under different temperatures are shown in Figure 3, and the grain size as shown in Table 1 was manually measured using the intercept method according to ASTM E112 [29]. For other thicknesses of sheets, only the temperature of 400 °C was selected to eliminate the effect of strain hardening. The heat treatment process was only used to eliminate the hardening effect during the manufacturing of the thin sheet with the rolling process. The difference of grain size for different thicknesses was ignored for the same heat treatment process. Thus, grain sizes of thin sheets with different thicknesses were not measured in this investigation.

## 3. Results and Discussion

### 3.1. Effect of UV on Properties of Specimen with Different Thicknesses

**Figure 4:**
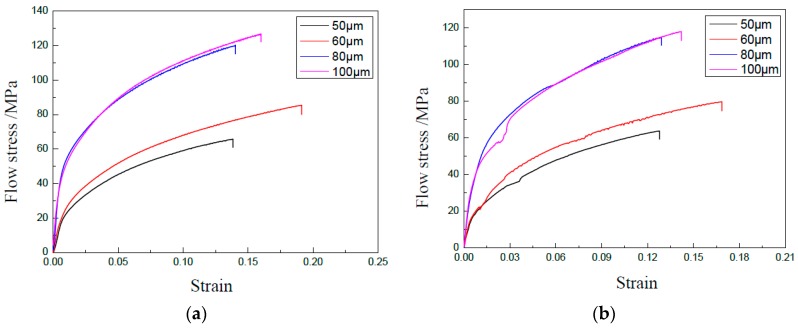
Curves of stress–strain of specimens with different thicknesses. (**a**) Without UV; (**b**) With UV.

During the miniaturization, size effects have an obvious influence on the properties of the specimen. To realize this kind of effects, GB 5052 specimens with different thicknesses were utilized after heat treatment on the temperature of 400 °C for 1 h, and uniaxial tensile tests were carried out with a punch speed of 0.3 mm/min and vibration amplitude of 0.48 μm. The experimental results are shown in Figure 4, demonstrating that the flow stress decreases with the decrease of thickness both without and with UV. This means that the size effect on flow stress occurs, which has been studied in many papers [2]. 

**Figure 5:**
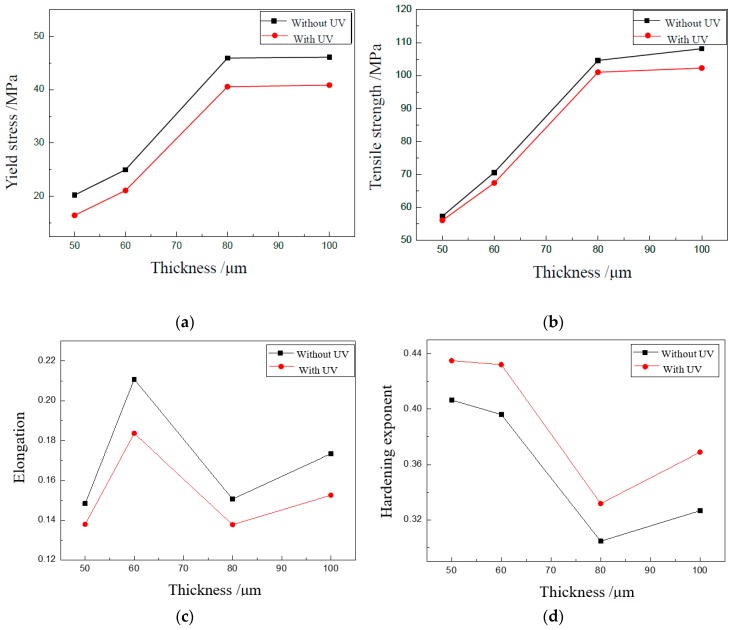
Effect of UV on properties of specimens with different thicknesses. (**a**) Effect of UV on yield stress; (**b**) Effect of UV on tensile strength; (**c**) Effect of UV on elongation; (**d**) Effect of UV on hardening exponent.

To study the effect of UV on the properties of materials in detail, the yield stress, tensile strength, elongation, and hardening exponent were achieved as shown in Figure 5 based on curves shown in Figure 4. It clearly shows that the thickness of the specimen has an obvious effect on the parameters of the thin sheet. The yield stress and tensile strength decrease with the decrease of thickness, and the elongation and hardening exponent increase with the decrease of thickness. Only the elongation for a thickness of 50 μm does not follow this rule, which becomes lower.

When UV was applied, the yield stress, tensile strength, and elongation decreased, and the hardening exponent increased, which was similar with the results shown above. For the acoustic softening effect, the flow stress can be reduced, since dislocations are activated by the energy and become easy to slip, even those on the ‘hard’ orientation. As a result, the yield stress and tensile strength become smaller in UV-assisted tests. When radial shrinkage occurs during tensile tests, UV can increase the extension of the crack since the acoustic energy density becomes bigger for the small cross-section area induced by the shrinkage. Then, the elongation is decreased by ultrasonic vibration. However, the proportion of uniform deformation is generally increased by UV, which is helpful in improving the critical limit. We plan to study these phenomena in future investigations.

An increase of the hardening exponent was observed in the tests by UV. The reason may be that UV activates more dislocations and promotes the interaction of dislocations. As a result, more subgrains are formed, which leads to the increase of the hardening exponent [27]

The effect of UV is almost the same for specimens with different thicknesses. This may be attributed to the fact that the acoustic energy is too small. The coupling effect of UV and dimension will be studied in depth in future investigations.

### 3.2. Effect of UV on Properties of Specimens with Different Grain Sizes

**Figure 6:**
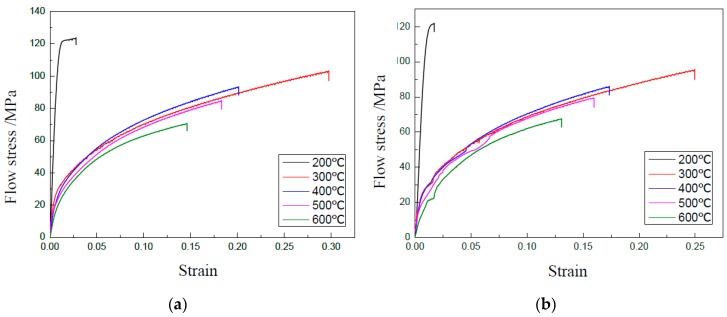
Curves of stress–strain of GB 5052 sheet. (**a**) Without UV; (**b**) With UV.

Uniaxial tensile tests were carried out with a punch speed of 0.3 mm/min and vibration amplitude of 0.48 μm using GB 5052 thin sheet specimens of 50 μm in thickness. The obtained curves of stress–strain for GB 5052 sheets with different grain sizes are shown in Figure 6 without and with UV, respectively. To analyze the effect of UV on the mechanical properties of GB 5052 thin sheet, yield stress, tensile strength, elongation, and harden exponent were obtained from the curves of stress–strain. 

**Figure 7:**
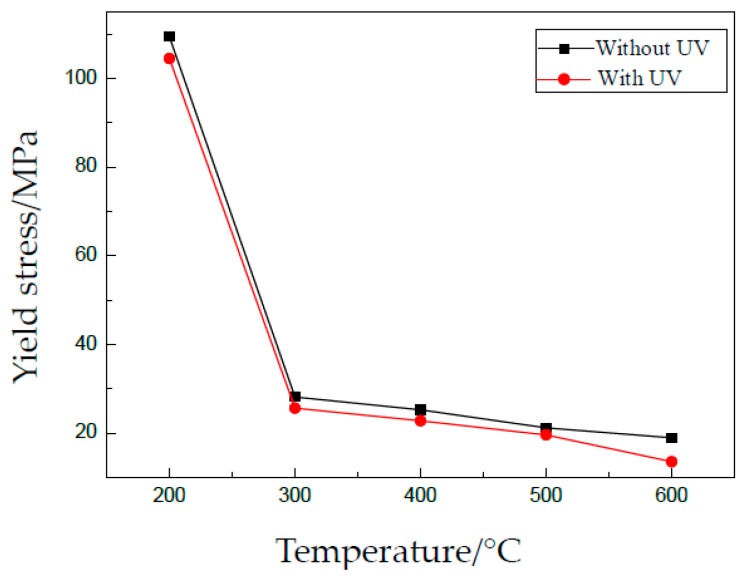
Effect of UV on yield stress.

**Figure 8:**
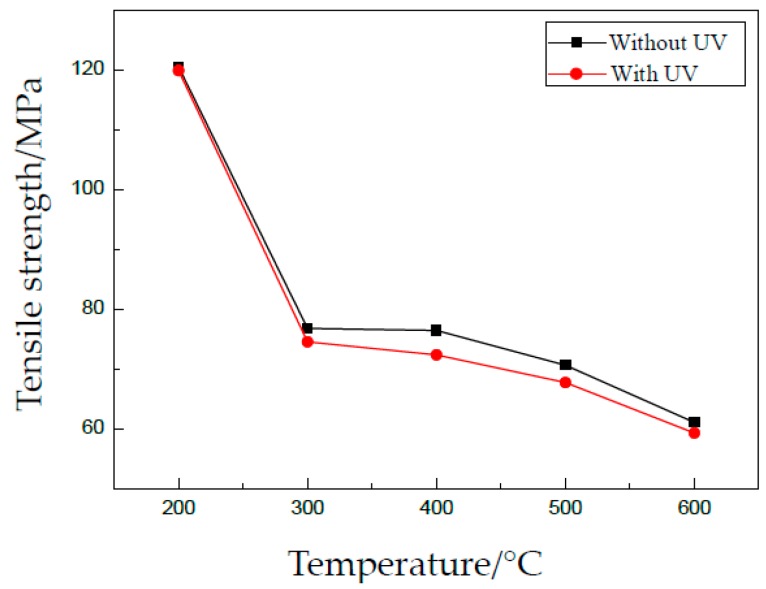
Effect of UV on tensile strength.

For the existing acoustic softening effect, the flow stress is reduced by UV. The yield stress and tensile strength are shown in Figure 7 and Figure 8, respectively. We notice that the data in Figure 8 are different from those in Figure 5c for the same condition. The reason may be that the tests were carried out at a different time and using different batches of material. 

A softening effect can be observed from the two figures, and the reduction of flow stress is very small. The reason is that the softening effect depends on the level of acoustic intensity applied on specimens. A little acoustic energy used in these experiments, which is proportional to the vibration amplitude, accordingly leads to a small reduction of flow stress. 

For different grain sizes, the effects of UV are almost similar. One reason is the small vibration amplitude. Another one, also important, is that the density of defects in thin sheets becomes very similar after heat treatment. Generally, defects in metallic materials can absorb acoustic energy and activate dislocations to move, which leads to a reduction of shear stress during plastic deformation. Thus, the effects of UV on yield stress and tensile strength are very small in this investigation. 

**Figure 9:**
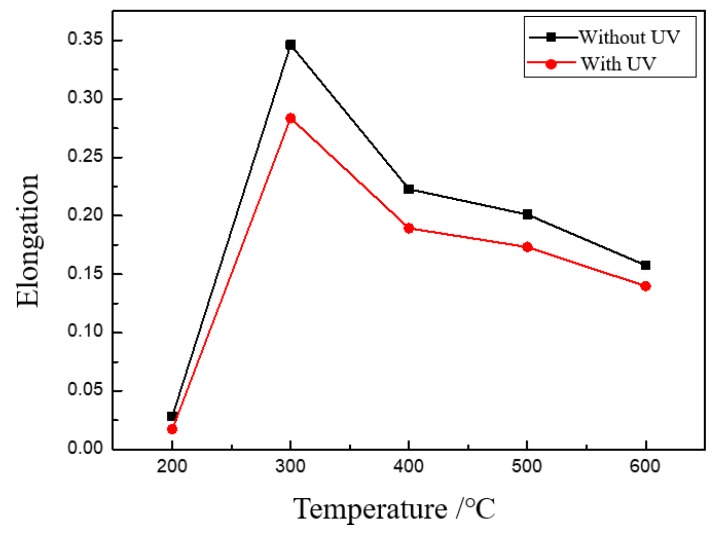
Effects of UV on elongation.

The elongation of thin sheets indicates the limited plastic deformation ability of metallic materials, which is very important in thin sheet forming. The effect of UV on the elongation of thin sheet with different grain sizes is shown in Figure 9. It is found that the elongation of the thin sheet is decreased, and the reduction slightly increases with the increases of grain size. During the uniaxial tensile deformation, radial shrinkage will occur before the fracture of the specimen. The appearance of radial shrinkage means that the area of the cross-section becomes smaller. With the same vibration amplitude, as well as the same input acoustic energy, the acoustic energy density will be increased for the smaller area, which will improve the activation of dislocations and local plastic deformation. As a result, the elongation with UV becomes smaller. 

**Figure 10:**
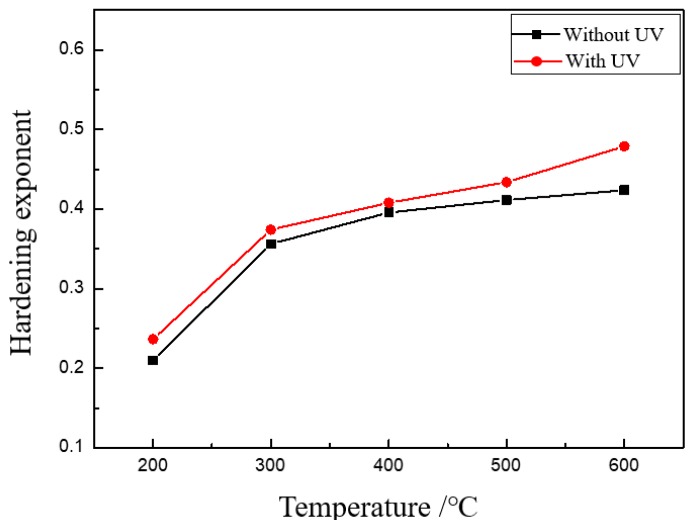
Effects of UV on the hardening exponent.

The hardening exponent is another important parameter which indicates the ability of uniform plastic deformation of thin sheet. The effect of UV on the hardening exponent is shown in Figure 10. The experimental results showed that UV increases the hardening exponent, especially for specimens with a bigger grain size, which means that the uniform deformation ability is increased by ultrasonic vibration. The reason is that UV as a kind of energy can activate more dislocations to move, even dislocations in an unfavorable position, which is validated by Electron Backscattered Diffraction (EBSD) analysis [25]. The more dislocations are activated, the more uniform plastic deformation can be realized. 

### 3.3. Effect of UV Duration on Properties of Specimen

**Figure 11:**
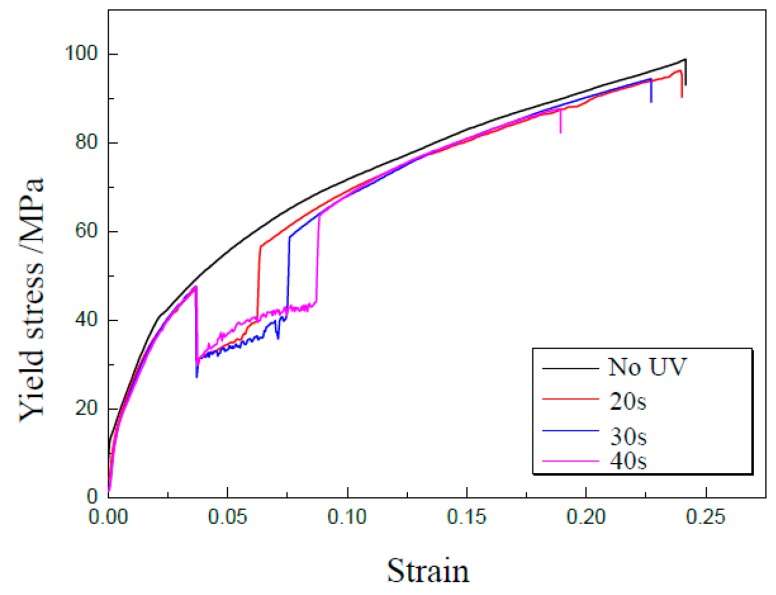
Effect of UV duration on flow stress.

The different duration of UV means a different acoustic energy applied on the specimen, and the effect of UV duration was analyzed in this investigation. With GB 5052 thin sheets of 50 μm in thickness treated on the temperature of 400 °C, UV-assisted uniaxial tensile experiments were carried out with a punch speed of 0.3 mm/min using a vibration amplitude of 5 μm under different durations of 20, 30, and 40 s, respectively. Experimental results showed that the flow stress can be decreased immediately during tensile deformation when UV is applied on the specimen as shown in Figure 11. This means that the acoustic softening effect occurs. The reduction of flow stress is almost the same for the same vibration amplitude. When UV is turned off, the flow stress goes back to that without UV. However, the flow stress becomes smaller after UV, which can be treated as a kind of residual effect because the acoustic energy forces dislocations to move in a preferred direction, and materials’ properties undergo permanent changes [24].

**Figure 12:**
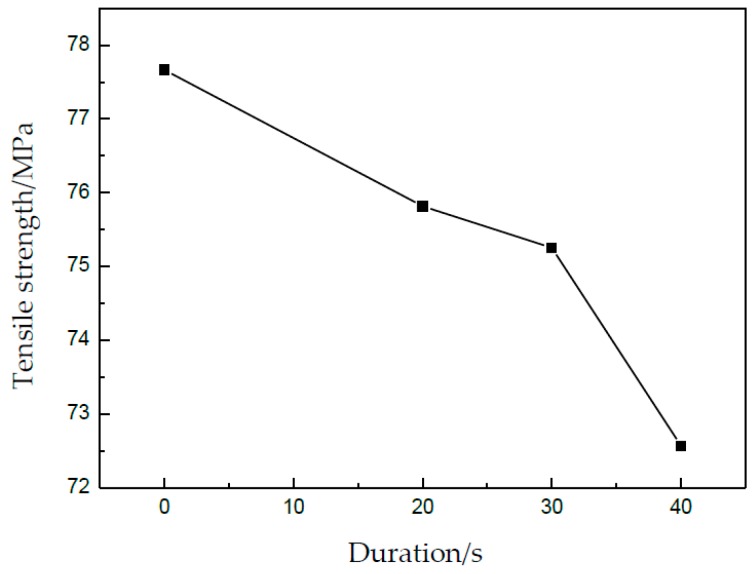
Tensile strength under different durations.

**Figure 13:**
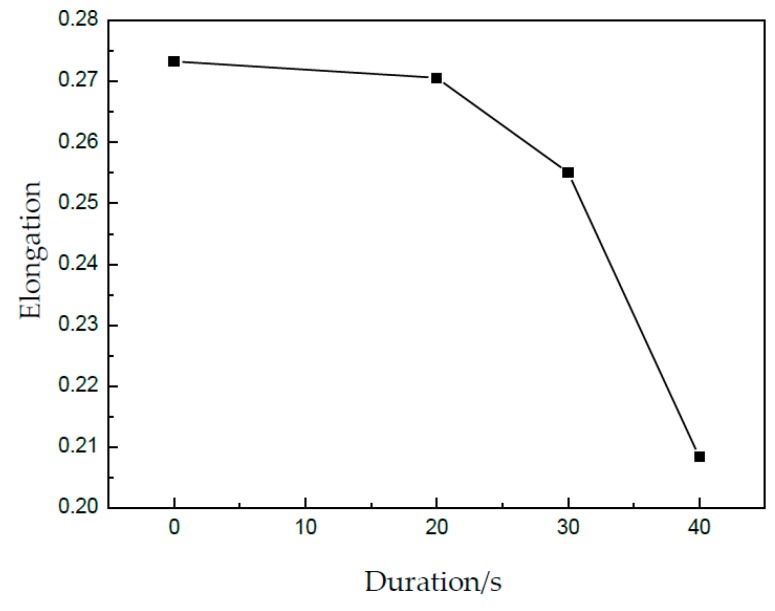
Elongation under different durations.

In Figure 11, the effect of UV duration on tensile strength and elongation can be obtained, as shown in Figure 12 and Figure 13, respectively. The tensile strength can be obviously decreased from 77.6 MPa for 0 s to 72.5 MPa for 40 s; the reduction percentage is about 6.6%. Further, the elongation was reduced from 0.73 for 0 s to 0.21 for 40 s. Although UV is turned off, its effect on the tensile strength and elongation is very clear, which is called a kind of residual effect. This may be attributed to the permanent changes of the microstructure in metallic materials after UV-assisted deformation [24]. For a longer duration of UV, more acoustic energy was applied on the specimen, and its effect on the properties increased accordingly.

### 3.4. Effect of Vibration Amplitude on Properties of Specimen

**Figure 14:**
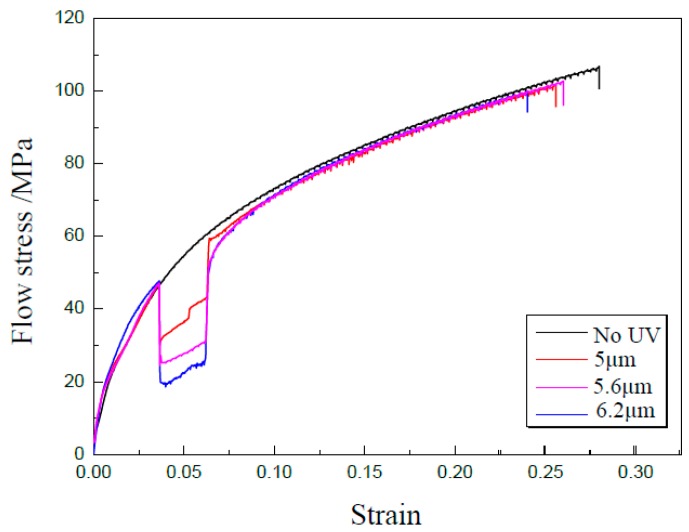
Curves of strain-stress under different vibration amplitudes.

As we know, acoustic energy is proportional to vibration amplitude, which has an obvious effect on the properties of materials. In this investigation, UV-assisted uniaxial tensile experiments were carried out with a punch speed of 0.3 mm/min using a duration of 30 s under different vibration amplitudes of 5, 5.6, and 6.2 μm, respectively. Further, the GB 5052 thin sheet of 50 μm in thickness treated on the temperature of 400 °C was selected, and the experimental results are shown in Figure 14. It was found that the flow stress decreases immediately. The reduction of flow stress was 16.43, 21.67, and 24.82 MPa, respectively. A higher level of acoustic intensity may provide more energy to motivate more dislocations to slide from their pinned points and also lead to a multiplication of dislocations [24]. As a result, the reduction of flow stress increases with the increase of vibration amplitude.

## 4. Conclusions

In this investigation, UV-assisted uniaxial tensile tests were carried out utilizing GB 5052 thin sheets. The effects of UV on the properties of materials were analyzed in detail for specimens with different grain sizes and original thicknesses, respectively. An acoustic softening and residual effect was found in the investigation. The following conclusions can be obtained.

1. Flow stress, e.g., yield stress and tensile strength, is decreased for the acoustic effect. Additionally, the reduction of flow stress increases with the increase of vibration amplitude. The reason is that acoustic energy, which is proportional to amplitude, activates dislocations and gives the energy for dislocation slipping.
2. Since lots of dislocations, even ‘hard’ orientation, are activated by acoustic energy, the interactive effects of dislocations are increased. Then, the hardening exponent becomes bigger, which can improve the uniform deformation ability of thin sheets.
3. With the increased duration of UV, tensile strength and elongation are decreased. Even when UV is turned off, its effect is still observed, which is called a residual effect. The reason may be that the microstructure of the specimen is permanently changed by UV.
4. The effect of UV on the properties of specimens with different grain sizes and thicknesses is similar. The reason may be that the acoustic energy is small. The coupling effect of UV and specimen dimension will be studied in future investigations.

## Figures and Tables

**Table 1:** Grain size of a GB 5052 thin sheet after annealing treatment (50 μm in thickness).

**Temperature/°C**	200	300	400	500	600
**Grain Size/μm**	4.3	13.68	18.7	25.55	47.65
